# Patient-Derived Xenograft Mouse Model of a Rare Gynecologic Malignancy: Personalized Medicine for the Treatment of Mesonephric-Like Adenocarcinoma

**DOI:** 10.7759/cureus.95530

**Published:** 2025-10-27

**Authors:** Kanako Kasuya, Yasuto Kinose, Yan Wang, Mai Koizumi, Aasa Shimizu, Mahiru Kawano, Michiko Kodama, Eiji Kobayashi, Kenjiro Sawada, Tadashi Kimura

**Affiliations:** 1 Department of Obstetrics and Gynecology, the University of Osaka Graduate School of Medicine, Suita City, JPN; 2 Department of Obstetrics and Gynecology, Oita University Faculty of Medicine, Yufu City, JPN

**Keywords:** mesonephric-like adenocarcinoma, omipalisib, patient-derived xenograft, precision medicine, trametinib, uterine cancer

## Abstract

Background: Mesonephric-like adenocarcinoma (MLA) is a rare malignant tumor that mainly occurs in the uterine body and ovaries and has characteristics similar to mesonephric adenocarcinoma in the uterine cervix. Although MLA has a poor clinical prognosis, no standard treatment for MLA has been developed, mainly because of its rarity. Here, we aimed to develop a precision medicine platform for MLA using a patient-derived xenograft (PDX) mouse model.

Methods: We established an MLA PDX mouse model via orthotopic implantation of a primary uterine tumor. Whole exome sequencing of the primary MLA tumor was performed to detect the genomic characteristics. For drug testing, we generated a patient-derived explant (PDE) model using sliced PDX tumors on medium-soaked gelatin sponges exposed to drugs. As another evaluation method, we cultured MLA cells from PDX tumors via a three-dimensional culture method with each drug treatment. Furthermore, we investigated the antitumor effects of a combination of trametinib and omipalisib compared with carboplatin in MLA PDX mice in vivo.

Results: MLA PDX tumors exhibited similar histological features to the original patient tumors. Whole exome sequencing revealedpathogenic variants of*KRAS* and *PIK3CA* in the patient's tumor. In PDE and three-dimensional culture models, carboplatin, paclitaxel, trametinib, and omipalisib had antitumor effects on MLA compared with the control. In addition, the combination of trametinib and omipalisib significantly suppressed MLA PDX tumor growth compared with the control.

Conclusions: We established a PDX model of MLA to facilitate personalized treatment for rare tumors.

## Introduction

Recently, with the rapid progress of science and technology, we have come to understand the genomic abnormalities that are the essence of cancer, and many kinds of comprehensive genomic profiles of cancer have been put into practical use in clinical settings, strongly promoting personalized cancer medicine. However, at present, even when genetic mutations that can be linked to potential drugs are detected via genomic tests, actual treatment is carried out in only a small number of cases, and this is an urgent issue that needs to be overcome in current cancer treatment [1]. Therefore, there is a strong need for research and development of truly individualized cancer medicine that can be applied in real-world clinical practice.

Mesonephric adenocarcinoma (MA) is a rare malignant neoplasm that occurs mainly in the cervix and is HPV-independent, with differentiation into the mesonephric duct [2]. Following the recent revision of the WHO classification of gynecological tumors, mesonephric-like adenocarcinoma (MLA) was classified as a rare tumor that presents characteristics such as MA in the uterine corpus and the ovary [2]. Both MA and MLA are thought to originate from a mesonephric origin [2], whereas some researchers suggest that MLA arises from the Müllerian epithelium with mesonephric differentiation. Although there are no large-scale epidemiological data on MLA yet, several studies have reported that MLAs are more likely to be diagnosed at an advanced stage than other histological types are and that the rate of recurrence of lung metastases after treatment is relatively high [3, 4]. Thus, MLA is regarded as a poorer prognostic histotype compared with grade 1/2 endometrioid endometrial cancer under current clinical standard treatment. *KRAS *and *PIK3CA* mutations are commonly found in approximately 85% and 20% of MLAs of the female genital tract, respectively [3-7]. Surgical treatment of total hysterectomy and bilateral salpingo-oophorectomy with or without adjuvant platinum-based chemotherapy is often performed similarly to other histotypes of gynecologic malignancies [8]. To the best of our knowledge, because of its rarity, there are no commercially available cell lines or in vitro and in vivo research models for MLA. In addition, owing to the small number of cases, it is difficult to conduct clinical trials, and no standard treatment for MLA has been established.

Many tumor models have been used to elucidate the mechanisms of cancer development and biology, as well as to develop treatments. Cancer cell lines are widely used in research because they are generally inexpensive and easy to culture and expand in vitro. However, drastic changes in genome characteristics and gene/protein expression might have occurred after the establishment of cell lines following long periods of culture and many passages, resulting in the inability of the cells to retain the original characteristics of the tumor [1]. Regarding this concern, only approximately 5% of the anticancer drugs that have demonstrated efficacy in preclinical studies using cell lines have been approved by the U.S. Food and Drug Administration. On the other hand, a patient-derived xenograft (PDX) is an experimental model that is established by xenotransplantation into immunodeficient animals, and the original human tumors are allowed to grow and pass through, maintaining the characteristics of the patient’s tumors. PDX mouse models maintain the heterogeneity and three-dimensional (3D) structure of human tumors, and PDX tumors recapitulate histological findings, genomic abnormalities, and protein expression. The use of PDX mouse models has promoted the elucidation of biology and the development of therapeutic drugs in the clinic, including gynecological oncology, in recent years [9-11]. The antitumor effects of PDX mouse models, which faithfully reproduce the structure and properties of human tumors, are suggested to strongly correlate with clinical efficacy in humans compared with conventional animal experiments using cell lines. For this reason, research results using PDXs are strongly recommended when basic research results are applied to human clinical trials.

In this study, we established an MLA PDX mouse model via orthotopic transplantation of a human primary tumor and a personalized medical platform for MLA via PDX tumors.

## Materials and methods

Patient sample collection and ethical approval

Written informed consent was signed after the patient received an explanation of the research, including genomic analysis. This study was approved by the University of Osaka (Suita, Osaka, Japan) ethical committee (approval number #873-2). All animal experiments were performed with the approval of the Institutional Animal Care and Use Committee of the University of Osaka (approval number #02-014-006) in accordance with institutional and NIH guidelines.

PDX model

Female BALB/cSlc-nu/nu mice were purchased from Japan SLC, Inc. (Hamamatsu, Shizuoka, Japan). A PDX model (OU-UT3) was established from the uterine parametrial tumor of a 63-year-old female who underwent total abdominal hysterectomy and bilateral salpingo-oophorectomy at the University of Osaka Hospital (Suita, Osaka, Japan).

The patient’s tumors were collected during surgery and stored in cold wash buffer on ice. The wash buffer contained HBSS(-) without Ca, Mg and phenol red (Nacalai Tesque Inc., Kyoto, Japan, #17461-05), 2% fetal bovine serum (Sigma‒Aldrich Co., St. Louis, MO, #173012), 10% Pen/Strep (Nacalai Tesque Inc., #26252-94), 2.50 µg/ml amphotericin B (Nacalai Tesque Inc., #02743-04) dissolved in DMSO (Nacalai Tesque Inc., #13407-45) stock solution, and 0.1 mg/ml primocin (InvivoGen, San Diego, CA, #ant-pm-05) dissolved in DMSO.

The tumors were cut into 2-3 mm^3^ pieces, some of which were fixed in 10% neutral buffered formalin (FUJIFILM Wako Pure Chemical Corporation, Osaka, Japan, #062-01661) for 24 hours or preserved at -80°C for genomic analysis. For future in vivo implantation, several viable tumor chunks were gradually frozen in 7% DMSO at -80°C and stored in liquid nitrogen. 

Tumor chunks were orthotopically implanted onto the left ovary and distal to the left uterine horn of nude mice with 3-0 polyester sutures (Natsume Seisakusho Co., Tokyo, Japan, #F25-30T1, 70 cm) under general anesthesia using isoflurane (FUJIFILM Wako Pure Chemical Corporation, #099-06571) combined with the subcutaneous injection of butorphanol (Meiji Animal Health Co., Kumamoto, Japan, #804Y2283) as a pain killer. Before closure of the peritoneum and skin, the sutured tumors were covered with 200 µl of Matrigel (Corning Inc., NY, #356234).

The mice were assessed daily for general health and weighed weekly. Tumor growth was confirmed by palpation and measurement via transabdominal ultrasound. The tumor volume was calculated as tumor length × tumor width^2^ × 1/2. When the PDX tumors were greater than 1000 mm³in size, the mice were euthanized, and the tumors were harvested. Some pieces of the PDX tumors were used for drug tests, and others were utilized for implantation to expand tumor samples or preservation, as described above.

Hematoxylin and eosin (HE) staining and immunohistochemistry (IHC)

Formalin-fixed, paraffin-embedded blocks of the patient’s tumor or the PDX tumors were cut into 5 µm sections. The slides were deparaffinized in xylene and rehydrated with a graded ethanol series. HE staining was performed according to standard protocols. Immunohistochemical antigen unmasking was performed by boiling in Target Retrieval Solution (pH 9.0; Nichirei Biosciences Inc., Tokyo, Japan, #415201) for 25 minutes. After blocking with blocking solution (Agilent Technologies, Inc., Santa Clara, CA, #S2023), the slides were incubated with primary antibodies against GATA3 (1:400, Cell Signaling Technology, Danvers, MA, # 5852), ER (1:200, Abcam, Cambridge, UK, #ab16660), and CD10 (1:250, Cell Signaling Technology, #65534) overnight at 4°C. The samples were incubated with a primary antibody against Ki-67 (1:400, Cell Signaling Technology, #9027) for 30 minutes at room temperature. Thereafter, the slides were washed with Tris-buffered saline with 0.05% Tween-20, incubated with N-Histofine Simple Stain MAX PO (MULTI) (Nichirei Biosciences Inc., Tokyo, Japan, #414151F) for 30 minutes, and stained with DAB chromogen (Agilent Technologies, Inc., #K3467) for five minutes. Finally, the slides were counterstained with Mayer’s hematoxylin solution for four minutes.

Whole exome sequencing (WES) and analysis

Genomic DNA was extracted from frozen patient tumor samples via a DNeasy Blood & Tissue Kit (Qiagen, Hilden, Germany, #69504) according to the manufacturer’s protocol. WES and data analysis were performed at the NGS core facility of the Genome Information Research Center at the Research Institute for Microbial Diseases of the University of Osaka (Suita, Osaka, Japan). The DNA was hybridized to a set of custom-designed capture probes (Twist Comprehensive Exome Panel, Twist Bioscience, San Francisco, CA #102031). Sequencing was performed using a DNBSEQ-G400 (MGI Tech Co., Ltd., Shenzhen, China). Sample reads were aligned to the hg19 reference genome via BWA [12]. Single-nucleotide polymorphisms were called by Mutect2 [13]. Annotation was performed via ANNOVAR [14]. For filtering, we removed untranslated regions and synonymous variants and selected variants with minor allele frequencies <0.005 reported in the ToMMo database [15], 1000 Genomes Project [16], gnomAD [17], esp6500 [18], and HGVD [19]. Furthermore, we selected variants indicated as “likely pathogenic” or “pathogenic” by InterVar [20] or ClinVar [21].

Patient-derived explant (PDE) model

PDE models were constructed as previously reported [22, 23]. PDX tumors were dissected into 3 mm³ pieces and placed on top of a medium-soaked gelatin sponge (LTL Pharma Co., Ltd., Tokyo, Japan, #873322) in 24-well plates. The gelatin sponge was soaked with 1 ml of RPMI 1640 (Nacalai Tesque, #30264-85) containing 10% FBS, 1% P/S, 0.01 mg/mL hydrocortisone (Nacalai Tesque, #18403-51), and 0.01 mg/mL insulin (Nacalai Tesque, #12878-86). After the explants were cultured at 37°C and 5% CO2 for 24 hours, the drug agent or control (1% DMSO) was added to the medium. The drugs and concentrations were tested as described below. Paclitaxel (0.5, 5, 50 nM, Sigma‒Aldrich Co., #T7402), carboplatin (0.5, 5, 50 µM, FUJIFILM Wako Pure Chemical Corporation, #033-25231), cisplatin (0.5, 5, 50 µM, FUJIFILM Wako Pure Chemical Corporation, #15663-27-1), doxorubicin (1, 10, 100 nM, Sigma‒Aldrich Co., #25316--40-9), trametinib (1, 10, 100 nM, Selleck Chemicals, Houston, TX, #S4484), omipalisib (1, 10, 100 nM, Selleck Chemicals, #S2658), and a combination of paclitaxel (0.5 nM) and carboplatin (10 µM) were used. After being treated with agents for 72 hours, the explants were harvested, fixed, and embedded in paraffin for HE stains and IHC analysis as described above. The percentage of the proliferation marker Ki-67 was evaluated under a 40x microscope. Each experiment was performed with at least three explants.

Short-term 3D culture of cancer cells sorted from PDX tumors

The collected PDX tumors were chopped mechanically, placed in a 15 ml tube with 0.56 U/ml Liberase DH (Sigma‒Aldrich Co., #5401054001), and shaken in a water bath at 37°C for 1 hour. After the cells were filtered through a 40 µm cell strainer (Greiner AG, Kremsmünster, Austria, #542040), the cell suspension was centrifuged at 1000 × g for 3 minutes. After the supernatant was aspirated, the cell pellet was resuspended in cold 1% phosphate-buffered saline (Nacalai Tesque, #14249--95), and the number of cells was counted. Human-origin cells were separated from mouse-origin cells via a magnet-based mouse cell depletion kit (Miltenyi Biotec, Bergisch Gladbach, Germany, #130-104-694) according to the manufacturer’s protocol.

The cell pellets in the Matrigel domes on a 96-well plate were soaked in 3D culture medium and incubated at 37°C and 5% CO2. The 3D culture medium consisted of 50% conditioned medium from L-WNR cells (ATCC, Manassas, VA, #CRL-3276), 50% Advanced DMEM/F12 (Thermo Fisher Scientific, Inc., Waltham, MA, #12634010), 100× N2 supplement (Thermo Fisher Scientific, Inc., #17502048), 50× B27 supplement minus vitamin A (Thermo Fisher Scientific, Inc., #12587010), 100× GlutaMAX (Thermo Fisher Scientific, Inc., #35050061), 50 ng/ml recombinant human EGF (BioLegend, Inc., San Diego, CA, #585506), 500 nM ALK4, 5, and 7 kinase inhibitors (Abcam, #ab142092), 50 ng/ml recombinant human FGF-10 (BioLegend, Inc., # 559304), 50 ng/ml recombinant human HGF (BioLegend, Inc., #596402), 1 mM nicotinamide (Sigma‒Aldrich Co., #N0636), 1.25 mM N-Acretyl-L-citstein (Sigma‒Aldrich Co., #A9165), 1 nM estradiol (Sigma‒Aldrich Co., #E2758), and 10 μM Rho kinase inhibitor. The drugs and concentrations were tested as described below.

Paclitaxel (1, 10, 100 nM), carboplatin (1, 10, 100 µM), trametinib (10 nM, 100 nM, 1 µM), omipalisib (10 nM, 100 nM, 1 µM), and a combination of trametinib (10 nM) and omipalisib (10 nM) were used. We used 1% DMSO as a control. After exposure to each treatment for 72 hours, the adenosine triphosphate (ATP) levels of the 3D cultured cells were measured with a Cell-Titer-Glo viability assay (Promega Corporation, Madison, WI, #G9241). The relative light unit (RLU) was calculated by comparing the cell viability on day 0.

In vivo PDX preclinical test

When the tumor volume reached 100 mm³ after the PDX tumors were transplanted, the MLA PDX mice were randomized into three groups: control (n=5), carboplatin (n=3), and a combination of trametinib and omipalisib (n=6). Trametinib (2 mg/kg, Cayman Chemical Company, Ann Arbor, MI, #16292), omipalisib (0.25 mg/kg, MediChemExpress LLC., Monmouth Junction, NJ, #HY10297), and carboplatin (25 mg/kg, Tokyo Chemical Industry Co., Ltd., Tokyo, Japan, #C2043) were administered intraperitoneally twice per week, three times per week, or once/week, respectively. We checked the tumor volume and body weight each week. The mice were treated for seven weeks or euthanized due to humane endpoints, which were defined as weight loss >20% initial body weight, poor body condition score, or tumor volume > 2000 mm³.

Statistical analysis

All the data were analyzed with GraphPad Prism 10.2.1 (GraphPad Software, Boston, MA) and are expressed as the means ± standard errors unless otherwise indicated. Statistical analyses were performed via Dunn's multiple comparisons test or Dunnett’s multiple comparison test. P < 0.05 was considered statistically significant.

## Results

The PDX model maintained the pathological features of the original MLA tumor.

**Figure 1:**
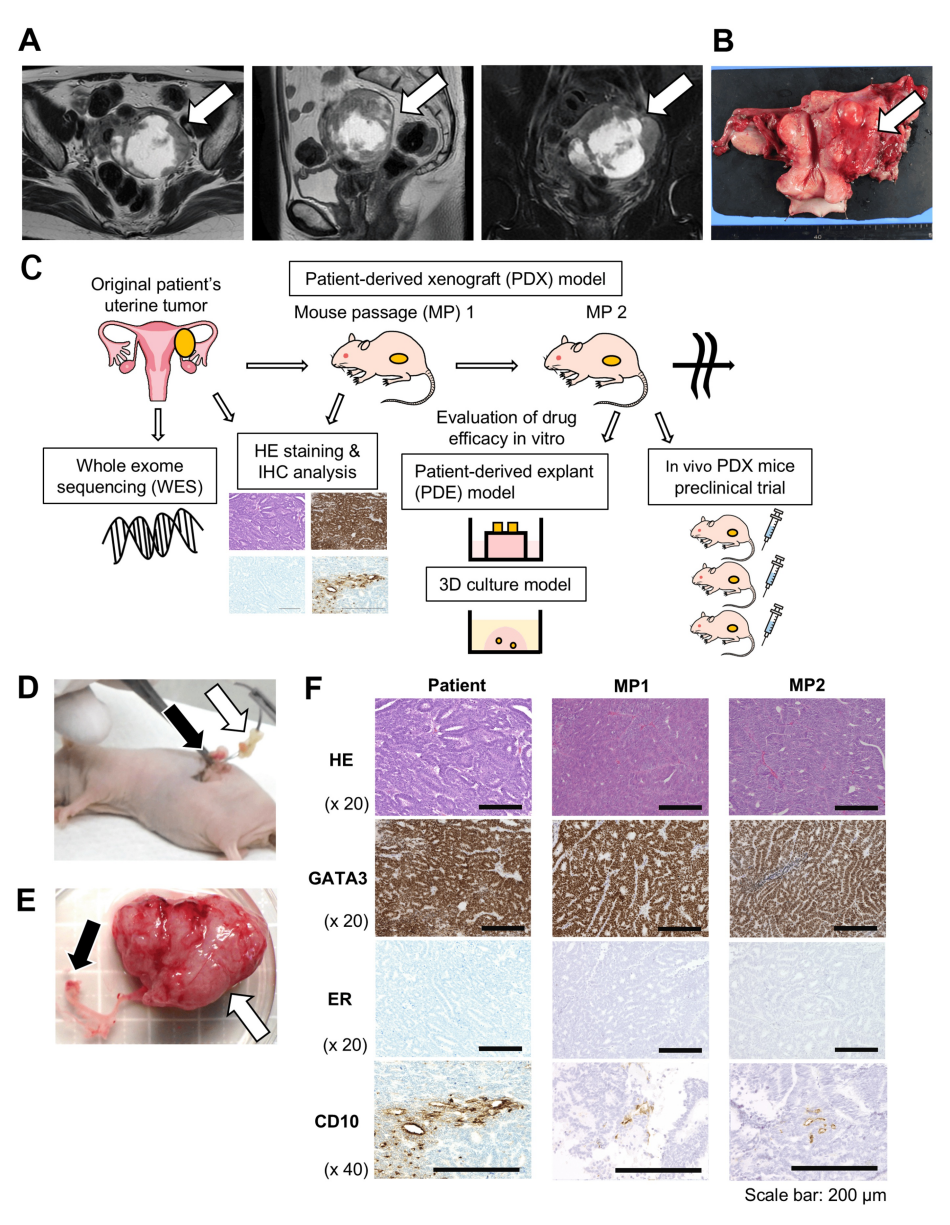
A patient-derived xenograft (PDX) model maintained the pathological features of the original mesonephric-like adenocarcinoma (MLA). (A) Magnetic resonance imaging of a patient with uterine MLA. Left: T2-weighted fast spin‒echo axial image. Middle: T2-weighted fast spin‒echo sagittal image. Right: T2-weighted fat-suppressed fast spin‒echo coronal image. The arrows indicate the MLA tumor. (B) Gross image of the MLA tumor. The tumor (arrow) extended from the left broad ligament to the myometrium of the uterus. (C) Schematic of the workflow used in this study. Whole exome sequencing (WES) of the primary MLA tumor was performed to detect the genomic characteristics. The PDX mouse model was established with the original patient’s tumor to expand the tumor for subsequent drug efficacy tests. Hematoxylin‒eosin (HE) staining and immunohistochemistry (IHC) of the original tumor and PDX tumor were performed. For the in vitro drug efficacy test, a patient-derived explant (PDE) model and a 3D culture model were used. For the in vivo drug efficacy test, PDX mice were used. (D) Orthotopic transplantation of the MLA tumor to generate a PDX mouse with a small incision in the left flank under general anesthesia. The white arrow indicates pieces of the tumor on a needle. The black arrow shows the left ovary and the distal end of the uterine horn of a nude mouse. (E) A picture of a PDX tumor harvested from a mouse. The white arrow indicates the PDX tumor on the left adnexa and the uterine horn, whereas the black arrow indicates a normal right ovary and a uterine horn. (F) HE and IHC images of original MLA tumors and PDX tumors. PDX tumors maintain protein expression similar to that of the original tumor. MP1: mouse passage 1, MP2: mouse passage 2, ER: estrogen receptor.

We collected an MLA tumor from the 63-year-old female patient during surgery. Preoperative magnetic resonance imaging revealed a 7.8 cm intrapelvic cystic mass with solid lesions (Fig. 1A). The pathological diagnosis was MLA arising from the uterine corpus and left broad ligament (Fig. 1B). Fig. 1C shows the workflow of this research using MLA tumors and a PDX model. We orthotopically transplanted the patient's MLA tumor into five immunodeficient mice (mouse passage 1: MP1) (Fig. 1D). One mouse died during the transplantation surgery due to deep anesthesia. After a few months, the MP1 tumor orthotopically implanted onto the left ovary and distal to the left uterine horn of the mouse grew over 1000 mm^3^ (Fig. 1E). Next, we generated 10 PDX mice (mouse passage 2: MP2) from MP1 tumors from three mice with rapidly growing PDX tumors. The PDX tumors developed in 100% (4/4) of the MP1 mice and 70% (7/10) of the MP2 mice. On average, MP1 and MP2 tumors were collected at 115 and 120 days after transplantation, respectively. We used PDX mice within eight passages in this research project. The histological findings revealed that MLA PDX tumors presented a similar histology to the original patient tumors (Fig. 1F). The tumors showed tubular and ductal patterns with HE stains, strong positive staining for GATA3, negative staining for estrogen receptor, and luminal staining patterns of CD10 with IHC. The total success rate of PDX tumor development was 71% (46/65) (MP3 (71%, 10/14), MP4 (56%, 5/9), MP5 (38%, 3/8), MP6 (60%, 3/5), MP7 (100%, 10/10), and MP8 (80%, 4/5)).

Pathogenic somatic *KRAS *and *PIK3CA *mutations were identified via WES of the original MLA tumor.

**Figure 2:**
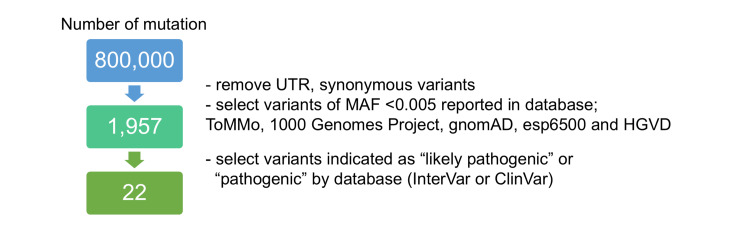
Workflow of whole-exome sequencing. Genomic DNA was extracted from frozen patient tumors. Approximately 800,000 genetic variants have been identified. For filtering, we removed untranslated regions (UTRs) and synonymous variants and selected variants with minor allele frequencies (MAFs) < 0.005 reported in ToMMo, the 1000 Genomes Project, gnomAD, esp6500, and HGVD. Thereafter, we sorted the variants indicated as “likely pathogenic” or “pathogenic” by InterVar or ClinVar. As a result, 22 single nucleotide variants were identified.

To investigate the therapeutic target of the MLA tumor, we performed WES on the patient’s original tumor (Fig. 2). After in silico analysis, WES revealed 22 pathogenic genetic mutations in 11 genes (Table 1). Stop-gain mutations in six genes, nonsynonymous single-nucleotide variants in four genes, and splicing mutations in one gene were identified. Among the variants, two nonsynonymous single-nucleotide variants,* KRAS* (exon 2:c.G35T:p.G12V, allele frequency 0.714) and* PIK3CA* (exon 2:c.G263A:p.R88Q, allele frequency 0.42), have been previously reported as pathogenic somatic mutations in cancers. On the basis of these results, we selected trametinib (MEK inhibitor, also known as mitogen-activated protein kinase kinase (MAPKK) inhibitor) and omipalisib (phosphoinositide 3-kinase (PI3K)/mammalian target of rapamycin (mTOR) dual inhibitor) as potential therapeutic drugs that target the downstream signals of *KRAS* and *PIK3CA*, respectively.

**Table 1:** Whole-exome sequencing revealed pathogenic KRAS and PIK3CA mutations in the MLA tumor. Whole-exome sequencing of the MLA tumor revealed 22 single-nucleotide variants in 11 genes. Among these variants, *KRAS* and *PIK3CA* have been previously reported as pathogenic somatic mutations in cancers.

Gene	Allele Frequency	Genetic Mutation
KRAS	0.714	exon2:c.G35T:p.G12V
SLC26A4	0.537	exon8:c.919-2A>G
PIK3CA	0.42	exon2:c.G263A:p.R88Q
PDE3A	0.048	exon12:c.C1754T:p.A585V, exon13:c.C2720T:p.A907V
IPO11	0.047	exon27:c.C2503T:p.R835X
MSH2	0.044	exon9:c.G1198T:p.E400X, exon12:c.G1768T:p.E590X, exon13:c.G1570T:p.E524X
CACNA1C	0.038	exon1:c.T9G:p.Y3X, exon6:c.T342G:p.Y114X, exon8:c.T1134G:p.Y378X
CCNL1	0.034	exon6:c.C562T:p.R188X, exon10:c.C1180T:p.R394X, exon11:c.C715T:p.R239X
SUMF1	0.028	exon3:c.C495G:p.H165Q, :exon4:c.C570G:p.H190Q
PSMC5	0.028	exon5:c.T375G:p.Y125X, exon6:c.T444G:p.Y148X, exon6:c.T420G:p.Y140X
OBSCN	0.017	exon50:c.G13174T:p.E4392X, exon61:c.G16045T:p.E5349X

MLA responded to cytotoxic agents and molecular targeted drugs in PDE models in vitro.

**Figure 3:**
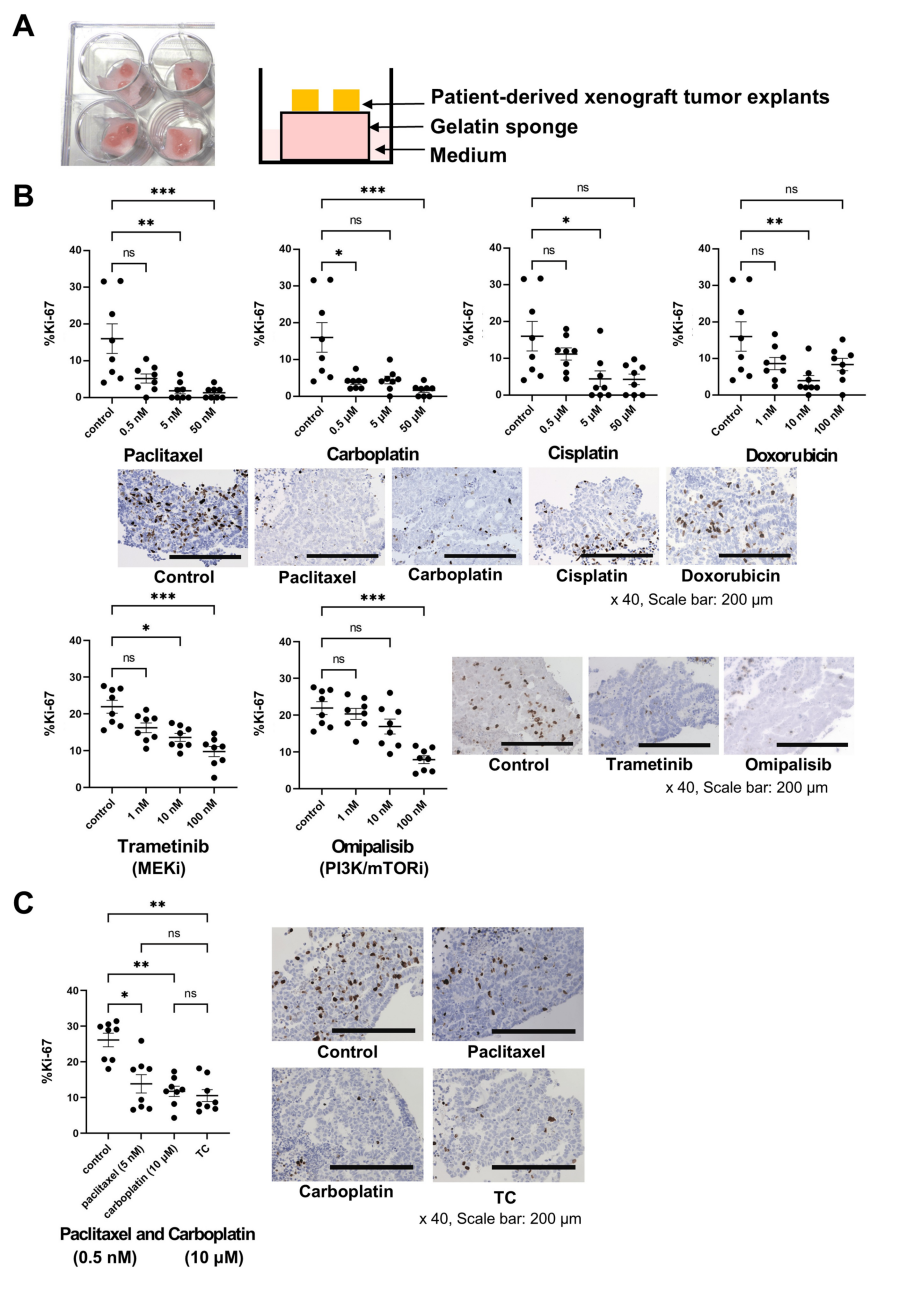
Drug response was evaluated in MLA patient-derived explant (PDE) MLA models. (A) Schematic of the drug test with the PDE model. PDX tumors were cut into 3 mm^3^ pieces and placed on a medium-soaked gelatin sponge (left; photo, right; schema). Each drug was added to the medium. After 72 hours of exposure, we harvested the tumor tissue. The antitumor response was assessed via immunohistochemistry with an anti-Ki-67 antibody. The percentage of Ki-67-positive cells was evaluated under a microscope (20x). (B) Results of the drug test using PDE models. Paclitaxel, carboplatin, cisplatin, doxorubicin, trametinib (MEK inhibitor), and omipalisib (PI3K/mTOR dual inhibitor) were evaluated. Each experiment was performed with at least three explants, and multiple views were assessed (n=8). The values represent the means ± standard errors. Statistical differences were examined by Dunn’s test. ns; not significant. * P<0.05. ** P<0.01. *** P<0.001. Representative immunohistochemistry images of PDE stained with anti-Ki-67 antibody. Highest dose group of each chemotherapy were presented. (C) Results of the drug test using PDE models. Paclitaxel, carboplatin, and a combination of paclitaxel and carboplatin were evaluated. Each experiment was performed with at least three explants, and multiple views were assessed (n=8). The values represent the means ± standard errors. Statistical differences were examined by Dunn’s test. ns; not significant. * P<0.05. ** P<0.01. Representative immunohistochemistry images of PDE stained with anti-Ki-67 antibody. TC; paclitaxel (0.5 nM) and carboplatin (10 μM).

With PDE models of MLA, we examined the effects of paclitaxel, carboplatin, cisplatin, and doxorubicin, which are generally used as cytotoxic agents in gynecology clinical practice, and trametinib (MEK inhibitor) and omipalisib (PI3K/mTOR dual inhibitor), which are potential molecular targeted drugs, which were selected on the basis of the WES results (Fig. 3A). We assessed antitumor responses via immunohistochemical staining of tumor explants for the proliferation marker Ki-67. Paclitaxel and carboplatin reduced the percentage of Ki-67-positive cells almost in a dose-dependent manner (%Ki-67: 50 nM paclitaxel; 1.32±0.55% vs. control; 16.03±4.02%, P<0.001, 5 nM paclitaxel; 1.85±0.84% vs. control; 16.03±4.02%, P<0.01, 50 μM carboplatin; 1.65±0.55% vs. control; 16.03±4.02%, P<0.001, 0.5 μM carboplatin 3.86±0.63% vs. control; 16.03±4.02%, P<0.05), suggesting that these widely used key drugs in gynecology also have antitumor effects on MLA (Fig. 3B). Cisplatin and doxorubicin worked as antitumor agents only at specific concentrations, and concentration-dependent effects are not observed. Compared with the control, trametinib and omipalisib reduced the percentage of Ki-67-positive cells in MLA explants at high concentrations (Ki-67: 100 nM trametinib; 9.74±1.38% vs. control; 21.94±1.76%, P<0.001; 100 nM omipalisib; 7.91±1.07% vs. control; 21.94±1.76%, P<0.001; Fig. 3B). Compared with the control, combination therapy with 5 nM paclitaxel and 10 μM carboplatin had significant antiproliferation effects but was not superior to either monotherapy alone (Ki-67: a combination of 5 nM paclitaxel and 10 μM carboplatin; 10.55±1.66% vs. controls; 26.1±1.91% (P=0.0017), vs. 5 nM paclitaxel monotherapy; 13.9±0.03% (not significant), vs. 10 μM carboplatin monotherapy; 11.8±0.01% (not significant), Fig. 3C).

Compared with the control, carboplatin, paclitaxel, omipalisib, and trametinib reduced the viability of MLA cells in in vitro 3D culture models.

**Figure 4:**
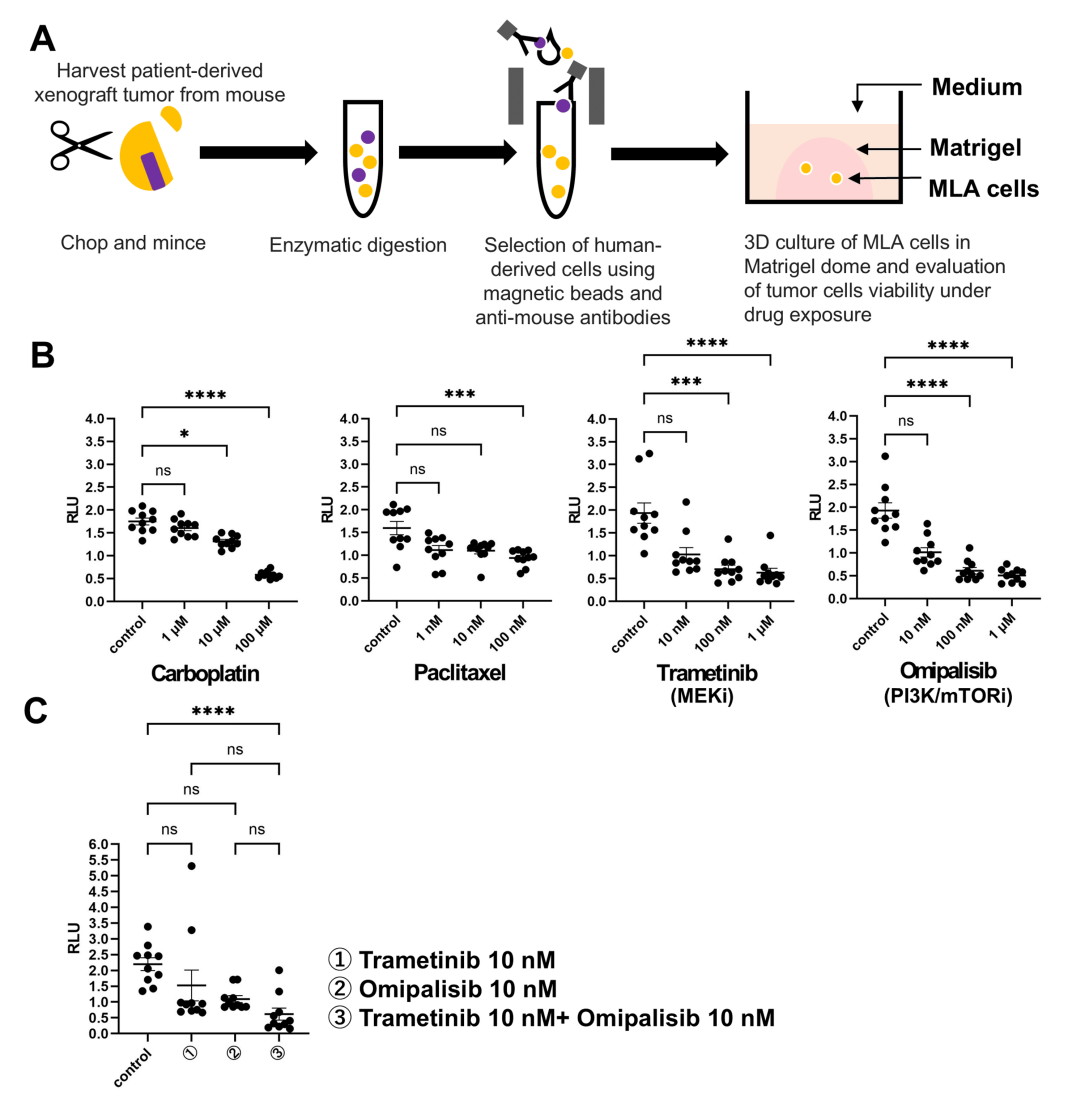
MLA represents the response to carboplatin, paclitaxel, trametinib, and omipalisib in three-dimensional (3D) culture models. (A) Schematic of the drug test with a 3D culture model using PDX tumors. The MLA PDX tumors were chopped, minced, and digested into single cells. Mouse-derived cells were excluded by an antibody-based magnetic separator. The MLA cells were cultured in Matrigel soaked in medium with supplements. The cells were exposed to drugs for 72 hours, and ATP levels, which indicate cell viability, were measured in relative light units (RLUs). (B) RLU level compared with that on day 0 (n=10) in each group. Monotherapy with paclitaxel, carboplatin, trametinib (a MEK inhibitor), or omipalisib (a PI3K/mTOR inhibitor) was tested. (C) A combination of trametinib and omipalisib was evaluated.Statistical differences were examined by Dunn’s multiple comparisons test. The data represent the means ± standard errors. ns; not significant. * P<0.05, *** P<0.001, **** P<0.0001.

We further evaluated the efficacy of the drug on MLA cells in a 3D culture model. Carboplatin, trametinib, and omipalisib caused a concentration-dependent decrease in ATP levels, indicating a concentration-dependent decrease in the viability of MLA tumor cells (RLU; 100 μM carboplatin; 0.58±0.02 vs. control; 1.75±0.08, P<0.0001, 1 μM trametinib; 0.62±0.10 vs. control; 1.93±0.22, P<0.0001, 1 μM omipalisib 0.51±0.05 vs. control; 1.93±0.17, P<0.0001; Fig. 4B). Compared with the control, a combination of 10 nM trametinib and 10 nM omipalisib significantly suppressed tumor cell viability, whereas each monotherapy was not superior to the control (RLU; 10 nM trametinib; 1.53±0.49 vs. control; 2.20±0.20, P=0.0908, 10 nM omipalisib; 1.09±0.11 vs. control; 2.20±0.20, P=0.1008, a combination of 10 nM trametinib and 10 nM omipalisib; 0.62±0.19 vs. control; 2.20±0.20, P<0.0001, 10 nM trametinib; 1.53±0.49 vs. a combination of 10 nM trametinib and 10 nM omipalisib; 0.62±0.19, P=0.2676, 10 nM omipalisib; 1.09±0.11 vs. a combination of 10 nM trametinib and 10 nM omipalisib; 0.62±0.19, P=0.2442, Fig. 4C).

A combination of trametinib and omipalisib suppressed MLA tumor growth in a PDX mouse model.

**Figure 5:**
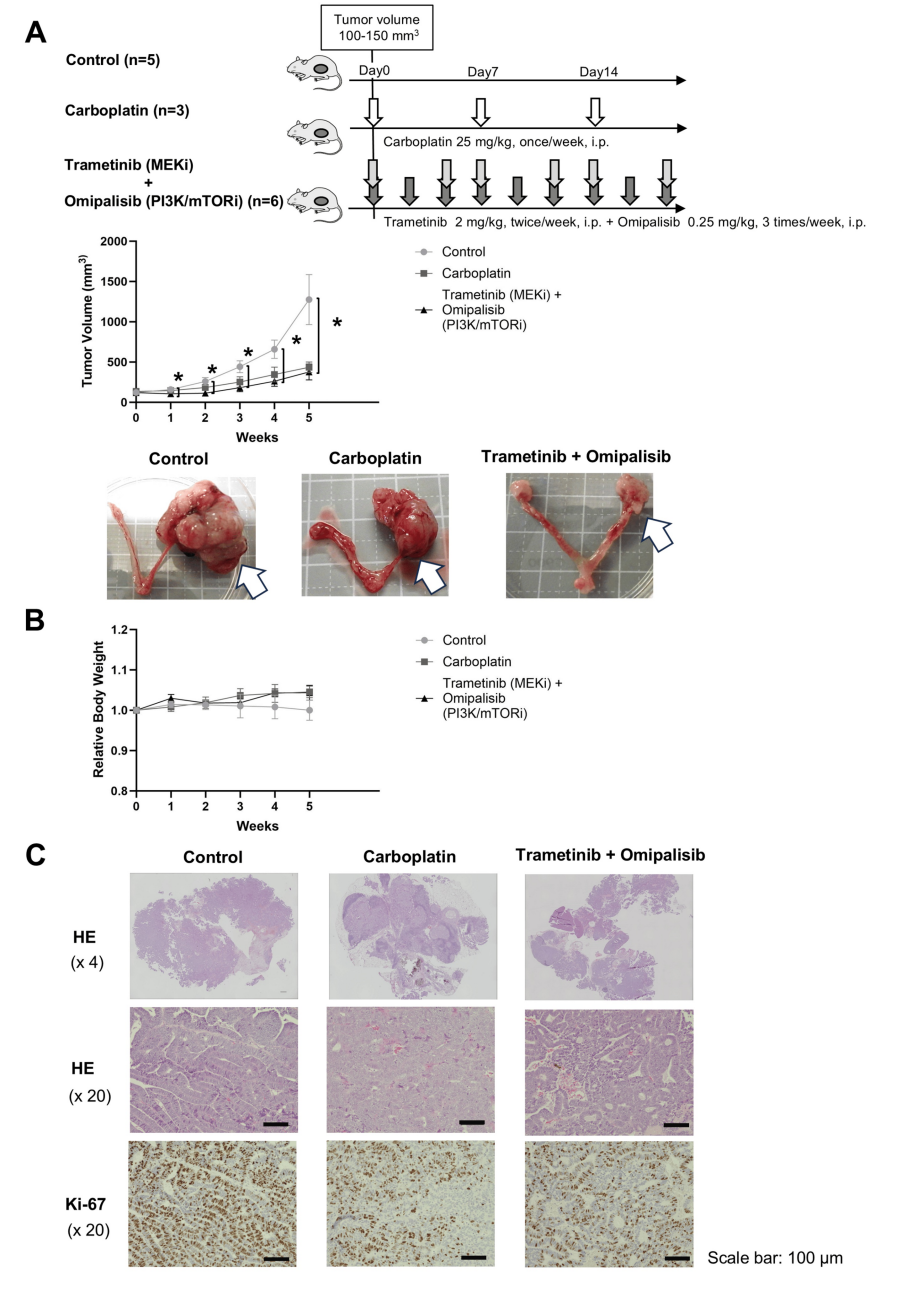
The combination of trametinib and omipalisib significantly suppressed MLA PDX tumors in vivo. (A) (upper) Schema of this in vivo study. When the PDX tumor volume reached 100 mm^3^, the mice were randomized into three groups: control (n=5), carboplatin (n=3), and the combination of trametinib (MEK inhibitor) and omipalisib (PI3K/mTOR inhibitor) (n=6).  PDX tumor volume, mouse body weight, and general condition were assessed weekly. (middle) Results of in vivo drug efficacy test with a MLA PDX mouse model. The values represent the means ± standard errors. Statistical differences were examined by Dunnett’s multiple comparisons test. * P<0.05. (lower) Representative images of tumors collected from each treatment group. The white arrows indicate the PDX tumor at the endpoint. (B) Relative body weight compared with that at week 0. No significant difference between the control and treatment groups was detected via Dunnett’s multiple comparisons test. (C) Histology and immunohistochemistry of MLA tumors from the PDX model in vivo were performed with an anti-Ki-67 antibody.

Finally, we examined the antitumor efficacy of carboplatin monotherapy and combination therapy with trametinib and omipalisib on MLA in a PDX mouse model. Compared with the control treatment, the combination of trametinib and omipalisib significantly reduced tumor size each week from the beginning of treatment (tumor volume at 5 weeks after the initiation of treatment; trametinib and omipalisib 377.9±98.6 mm^3^ vs. control; 1275.0±311.9 mm^3^, P<0.05; Fig. 5A). In contrast, although the tumor suppressive effect of carboplatin was observed to some extent, there was no significant difference in tumor volume between the carboplatin group and the control group at any point (tumor volume at five weeks after the initiation of treatment; carboplatin 435.2±66.3 mm^3^ vs. control; 1275.0±311.9 mm^3^, Fig. 5A). There were no significant differences in weight trends between the groups throughout the experimental period, and no severe weight loss was observed (Fig. 5B). The percentage of Ki-67-positive MLA PDX tumors tended to be greater in the control group than in the carboplatin and combination therapy groups, although this difference was difficult to quantify because of differences in tumor size and tumor/stromal areas (Fig. 5C).

## Discussion

We established a PDX mouse model using a patient-derived MLA tumor, a rare gynecological cancer. The PDX mouse model makes it possible to expand tumors stably and indefinitely and accurately preserves the histology, genomic abnormalities, and protein expression of human tumors. From the WES of the patient’s original tumor, we identified the *KRAS* and *PIK3CA* driver genes, which were reported to be frequently observed in MLA. We confirmed the antitumor effects of trametinib and omipalisib, which were identified as candidate drugs from driver genes, as well as conventional chemotherapeutic agents via in vitro tests (PDE models and 3D culture models) of PDX tumors. Furthermore, in vivo PDX mouse models revealed that the combination of trametinib and omipalisib had similar antitumor effects as the existing treatment carboplatin, and high tolerability was confirmed in mouse experiments.

To the best of our knowledge, this is the first PDX model of MLA that has been utilized to study personalized medicine. In the clinic, platinum-based chemotherapy is often performed empirically for MLA, in accordance with other histotypes of endometrial cancer and ovarian cancer [8]. Although reports on the treatment of recurrent MLA are limited, some studies have shown the therapeutic effects of the combination of carboplatin and paclitaxel to some extent [4, 24]. Our basic research in this study supports the clinical efficacy of the combination of carboplatin and paclitaxel on MLA. In addition, with the workflow using PDX tumors used in this study, we might be able to estimate the antitumor effect of conventional cytotoxic drugs in humans for other rare tumors for which we cannot obtain results from large-scale clinical trials.

A high KRAS mutation rate in MA and MLA has been previously reported [4-7]. A survey by da Silva et al. reported that 92% (13/14) of uterine MLAs and 87% (13/15) of ovarian MLAs harbor KRAS mutations [5]. KRAS mutation is often thought to be a potential treatment target, but few reports have used molecular-targeted drugs. Temsirolimus (an mTOR inhibitor) was used for the initial recurrence of uterine MLA, but the tumor volume increased by 10% [4]. In this study, we selected trametinib and omipalisib as treatments for MLA harboring KRAS and PI3CA mutations, respectively. Trametinib is a MEK1 and MEK2 inhibitor that is mainly used for melanoma with BRAF V600E or V600K mutations in clinical practice. The effects of trametinib on the inhibition of RAS signal transduction in KRAS-mutant tumors have been investigated. For example, trametinib showed a similar progression-free survival and response rate as docetaxel in patients with previously treated KRAS-mutant-positive non-small-cell lung cancer in a randomized phase II study [25]. Trametinib was associated with significantly improved PFS and ORR in patients with recurrent low-grade serous carcinoma, which is characterized by alterations in the MAPK pathway, compared to the physician’s choice standard of care [26]. Omipalisib is a PI3K/mTOR pathway inhibitor that targets the PI3K and mTOR pathways. One study reported that the inhibitory effect of omipalisib on the proliferation and migration of ovarian cancer cells, both in vitro and in vivo, was comparable to that of paclitaxel [27]. The safety profile of omipalisib has been investigated in a phase I study, and omipalisib is expected to be closely related to clinical application [28].

In many clinical trials, single-molecule targeted therapy has led to the emergence of resistant tumor cells within a short period of time. The combination of molecular target drugs is thought to help prevent tumors from acquiring resistance [29].

One phase I study of the combination of trametinib and omipalisib in patients with advanced solid tumors revealed one partial response with a KRAS-mutant ovarian cancer patient who remained in the study for >400 days at the time of data analysis, one stable disease with an ovarian cancer patient, and one progressive disease in patients with uterine endometrial cancer [30]. With respect to the toxicity of the combination of trametinib and omipalisib, skin- and gastrointestinal-related toxicities are frequently reported [30]. In our study, the low-dose combination of trametinib (MEK inhibitor) and omipalisib (PI3K/mTOR dual inhibitor) repressed tumor size in vivo for at least five weeks without significant adverse events and may be a durable treatment option for the original patient with MLA and other patients with MLA tumors harboring *KRAS* and *PIK3CA* variants. This kind of approach, combining genomic profiling and a PDX model, has the potential to facilitate personalized medicine as well as genome-based targeted cancer therapy.

For rare cancers such as MLA, especially if the prognosis is poor, there is an urgent issue of the lack of experimental and research models. This problem makes it more difficult to develop effective treatments for these malignancies than for common malignancies. Our orthotopic PDX models of gynecological cancers can be established with a high probability (71% in this study) on the basis of human tumors collected from surgery or biopsy and faithfully mimic the in vivo progression of cancer while accurately maintaining the characteristics of human ovarian and endometrial cancers. As mentioned above, the antitumor effects of drugs in PDX mouse models strongly correlate with the clinical response rate in humans. Hence, this research has the potential to significantly contribute to the development of personalized medicine for rare cancers. Furthermore, this type of research method can be applied not only to other rare gynecological cancers but also to rare cancer types across cancers.

There are several limitations in this study. First, only one MLA PDX model has been established because of the rarity of this cancer. By applying similar methods to facilities around the world, many PDX models can be created, even for rare cancers. In addition, multicenter studies using PDXs can be conducted to determine which drugs are truly effective for rare cancers in the clinic. Second, even if tumors grow rapidly, it usually takes two to three months after transplantation to harvest large PDX tumors over 1000 mm³. This means that it is difficult to identify immediately which drugs are genuinely effective in the clinical setting for patients with chemotherapy-resistant tumors. In this respect, maintaining and utilizing many PDX tumor banks with similar characteristics might enable us to deliver useful information for real-world clinical practice as quickly as possible. Third, further improvements in methods for PDX mice and drug testing should be developed in the future. In studies using immunodeficient mice, such as this study, it is difficult to evaluate the efficacy of immune checkpoint inhibitors, which have rapidly spread in clinical practice over the past decade. In vitro drug sensitivity tests also need to be improved further to determine which methods truly correlate with antitumor effects in humans more efficiently.

## Conclusions

In this study, we established a PDX mouse model of MLA, a rare gynecological cancer, and created a platform for the development of personalized medicine through in vitro drug sensitivity testing using PDX tumors and in vivo mouse experiments. On the basis of the workflow developed in this research, further studies are needed to investigate novel, effective treatments for malignant tumors that are resistant to current standard therapies, even if they are rare.

## References

[1] Mateo J, Steuten L, Aftimos P (2022). Delivering precision oncology to patients with cancer. Nat Med.

[2] WHO Classification of Tumours Editorial Board (2020). Female Genital Tumours. WHO Classification of Tumours, 5th Edition. Female Genital Tumours. WHO Classification of Tumours, 5th Edition.

[3] Na K, Kim HS (2019). Clinicopathologic and molecular characteristics of mesonephric adenocarcinoma arising from the uterine body. Am J Surg Pathol.

[4] Praiss AM, White C, Iasonos A (2024). Mesonephric and mesonephric-like adenocarcinomas of gynecologic origin: A single-center experience with molecular characterization, treatment, and oncologic outcomes. Gynecol Oncol.

[5] da Silva EM, Fix DJ, Sebastiao AP (2021). Mesonephric and mesonephric-like carcinomas of the female genital tract: molecular characterization including cases with mixed histology and matched metastases. Mod Pathol.

[6] Koh HH, Park E, Kim HS (2023). Mesonephric-like adenocarcinoma of the uterine corpus: genomic and immunohistochemical profiling with comprehensive clinicopathological analysis of 17 consecutive cases from a single institution. Biomedicines.

[7] Son J, Zhang Y, Lin H (2024). Clinical and genomic landscape of RAS mutations in gynecologic cancers. Clin Cancer Res.

[8] Deolet E, Van Dorpe J, Van de Vijver K (2021). Mesonephric-like adenocarcinoma of the endometrium: diagnostic advances to spot this wolf in sheep’s clothing. A review of the literature. J Clin Med.

[9] George E, Kim H, Krepler C (2017). A patient-derived-xenograft platform to study BRCA-deficient ovarian cancers. JCI Insight.

[10] Kim H, Xu H, George E (2020). Combining PARP with ATR inhibition overcomes PARP inhibitor and platinum resistance in ovarian cancer models. Nat Commun.

[11] Xu H, George E, Kinose Y (2021). CCNE1 copy number is a biomarker for response to combination WEE1-ATR inhibition in ovarian and endometrial cancer models. Cell Rep Med.

[12] Li H, Durbin R (2025). Fast and accurate short read alignment with Burrows-Wheeler transform. Bioinformatics.

[13] GATK GATK (2025). Broad Institute - GATK. Mutect2. https://gatk.broadinstitute.org/hc/en-us/articles/360037593851-Mutect2.

[14] Wang K, Li M, Hakonarson H (2010). ANNOVAR: functional annotation of genetic variants from high-throughput sequencing data. Nucleic Acids Res.

[15] Tohoku University Tohoku Medical Megabank Organization (2025). Tohoku University Tohoku Medical Megabank Organization. ToMMo [source in Japanese]. https://www.megabank.tohoku.ac.jp/.

[16] Auton A, Brooks LD, Durbin RM (2015). A global reference for human genetic variation. Nature.

[17] Karczewski KJ, Francioli LC, Tiao G (2020). The mutational constraint spectrum quantified from variation in 141,456 humans. Nature.

[18] Fu W, O'Connor TD, Jun G (2013). Analysis of 6,515 exomes reveals the recent origin of most human protein-coding variants. Nature.

[19] Higasa K, Miyake N, Yoshimura J (2016). Human genetic variation database, a reference database of genetic variations in the Japanese population. J Hum Genet.

[20] Li Q, Wang K (2017). InterVar: Clinical interpretation of genetic variants by the 2015 ACMG-AMP guidelines. Am J Hum Genet.

[21] Landrum MJ, Lee JM, Riley GR, Jang W, Rubinstein WS, Church DM, Maglott DR (2014). ClinVar: public archive of relationships among sequence variation and human phenotype. Nucleic Acids Res.

[22] Centenera MM, Hickey TE, Jindal S (2018). A patient-derived explant (PDE) model of hormone-dependent cancer. Mol Oncol.

[23] Mohammed H, Russell IA, Stark R (2015). Progesterone receptor modulates ERα action in breast cancer. Nature.

[24] Montagut C, Mármol M, Rey V (2003). Activity of chemotherapy with carboplatin plus paclitaxel in a recurrent mesonephric adenocarcinoma of the uterine corpus. Gynecol Oncol.

[25] Blumenschein GR Jr, Smit EF, Planchard D (2015). A randomized phase II study of the MEK1/MEK2 inhibitor trametinib (GSK1120212) compared with docetaxel in KRAS-mutant advanced non-small-cell lung cancer (NSCLC)†. Ann Oncol.

[26] Gershenson DM, Miller A, Brady WE (2022). Trametinib versus standard of care in patients with recurrent low-grade serous ovarian cancer (GOG 281/LOGS): an international, randomised, open-label, multicentre, phase 2/3 trial. Lancet.

[27] Xiao Y, Yu Y, Jiang P, Li Y, Wang C, Zhang R (2020). The PI3K/mTOR dual inhibitor GSK458 potently impedes ovarian cancer tumorigenesis and metastasis. Cell Oncol (Dordr).

[28] Munster P, Aggarwal R, Hong D (2016). First-in-human phase I study of GSK2126458, an oral pan-class I phosphatidylinositol-3-kinase inhibitor, in patients with advanced solid tumor malignancies. Clin Cancer Res.

[29] Aldea M, Andre F, Marabelle A, Dogan S, Barlesi F, Soria JC (2021). Overcoming resistance to tumor-targeted and immune-targeted therapies. Cancer Discov.

[30] Grilley-Olson JE, Bedard PL, Fasolo A (2016). A phase Ib dose-escalation study of the MEK inhibitor trametinib in combination with the PI3K/mTOR inhibitor GSK2126458 in patients with advanced solid tumors. Invest New Drugs.

